# Association between high biomarker probability of Alzheimer's disease and improvement of cognition and gait after shunt surgery in patients with idiopathic normal pressure hydrocephalus

**DOI:** 10.1186/2045-8118-12-S1-O48

**Published:** 2015-09-18

**Authors:** Hiroaki Kazui, Hideki Kanemoto, Yukiko Suzuki, Syunsuke Sato, Takashi Suehiro, Shingo Azuma, Kenji Yoshiyama, Toshihisa Tanaka

**Affiliations:** 1 Osaka University Graduate School of Medicine, Japan

## Introduction

In Alzheimer's disease (AD), the concentration of amyloid β1-42 (Aβ42) in cerebrospinal fluid (CSF) is low and that of total tau (t-tau) is high. We evaluated the relationship between high CSF biomarker probability of AD and improvement of cognition and gait after shunt surgery in idiopathic normal pressure hydrocephalus (iNPH).

## Methods

The subjects of this study were 37 iNPH patients (75.7±5.8 years, MMSE:22.2±4.2) who showed improvement of one of the triad symptoms at least after shunt surgery. We classified the patients into 16 patients with and 21 patients without the combination of low Aβ42 and high t-tau in CSF. We compared the improvement on cognitive and gait examinations 3 months after the shunt between the two groups with an analyses of covariance (ANCOVA), in which the score at 3 months after the shunt was entered in the model as a dependent variable, the baseline score as a covariate and the group as a categorical variable.

## Results

In 37 iNPH patients, significant improvement 3 months after shunt surgery was shown in the Timed Up and Go test (p<0.001), MMSE (p<0.001), attention/concentration of the WMS-R (p=0.028), and digit symbol substitution of the WAIS-III (p<0.001), but not in delayed recall of the short story in the Rivermead behavioral memory test (RBMT) (p=0.46). The ANCOVA revealed that the iNPH patients without high CSF biomarker probability of AD showed significantly greater improvement in the delayed recall of the RBMT 3 months after shunt surgery than those with high CSF biomarker probability of AD (p=0.017). In addition, in the latter group, the change 3 months after the shunt was not significant (p=0.14). The ANCOVA showed no significant differences in the improvement 3 months after the shunt surgery between the two groups on the other evaluations.

## Conclusion

The delayed recall ability might not improve after shunt surgery in iNPH patients with AD pathology.
